# 

**Published:** 1885-05

**Authors:** 


### BOOK NOTICES.

Our Bodies, or How We Live.—*By Albert F. Blaisdell, M. D.* This is one of the best elementary works on Physiology we have seen. It is written in a clear and simple style, in order to be readily understood by the young, since it is intended for use in schools. Special prominence is given to the subject of stimulants and narcotics. A novel idea is formed in the chapter on Practical Experiments. This part of the book is especially valuable, for by following out its instructions, the facts it teaches will be permanently fixed in the memory, a result so difficult to attain by text books alone. Lee & Shepard, Boston, Publishers.

Perils of American Women.—*By G. L. Austin, M. D.* This little book is, as its author has termed it, "A Doctor's Talk with Maiden, Wife and Mother." The author has taken for his subject the often repeated question, "What is the matter with American women?" He has treated a delicate subject in a sensible way, and without any of that cant of mock modesty which keeps so many of our women in ignorance of themselves and their functions; and yet he has carefully kept his treatise from being revolting to sensitive natures. Lee & Shepard, Boston, publishers.

= There will be issued by the New England Publishing Co., Sandy Hook, Conn., during the month of May, a book entitled "Berlin as a Medical Centre, by Horatio R. Bigelow, M. D., of Washington, D. C. This book will be a complete and accurate medical guide to Berlin, giving instructions in reference to board, clinics, lectures, expenses, etc. and all information that will be necessary for the medical student abroad. The price will be $2.00.

